# ALA Alleviates Liver Damage in Septic Mice Through PI3K/AKT Signaling Pathway

**DOI:** 10.1002/fsn3.70599

**Published:** 2025-07-10

**Authors:** Yuqing Song, Yuanrong Mao, Qingqing Sui, Zichen Zhao, Daosong Dong

**Affiliations:** ^1^ Department of Pain The First Hospital of China Medical University Shenyang Liaoning People's Republic of China; ^2^ Department of Pain Qinghai Provincial People's Hospital Xining Qinghai People's Republic of China

**Keywords:** alpha‐lipoic acid, antioxidant, apoptosis, PI3K/AKT, sepsis

## Abstract

Sepsis is a life‐threatening systemic inflammatory response syndrome that arises from infection and frequently progresses to multi‐organ dysfunction, with liver injury being a particularly significant manifestation. Alpha‐lipoic acid (ALA), renowned for its potent antioxidant and anti‐inflammatory properties, has demonstrated promising protective effects in sepsis. The present study aims to investigate the protective effects of ALA on liver damage in septic mice by modulating the PI3K/AKT signaling pathway, thereby providing novel therapeutic strategies for the treatment of sepsis. The cecal ligation and puncture (CLP) model was employed to induce sepsis in mice, and both ALA‐treated and control groups were established. The protective effects of ALA were evaluated through the detection of PI3K/AKT signaling pathway‐related protein expression, apoptosis in liver cells, and liver function indicators. Additionally, Western blotting and immunohistochemistry were utilized to further validate ALA's regulatory impact on the PI3K/AKT signaling pathway. The study revealed that ALA significantly enhanced the activation of the PI3K/AKT signaling pathway, reduced apoptosis in liver cells of septic mice, and improved liver function indicators. Moreover, liver tissue pathology in the ALA‐treated group was markedly less severe compared to that in the control group, indicating its effective hepatoprotective action. These findings demonstrate that ALA effectively alleviates liver damage in septic mice by promoting the PI3K/AKT signaling pathway, highlighting its potential clinical value. This research provides new targets and insights for the development of therapeutic strategies for sepsis.

## Graphical abstract

ALA improved survival and alleviated liver damage in septic mice. ALA exerted anti‐apoptotic and anti‐inflammation effects in mice with sepsis. ALA inhibited apoptosis through the PI3K/AKT pathway.

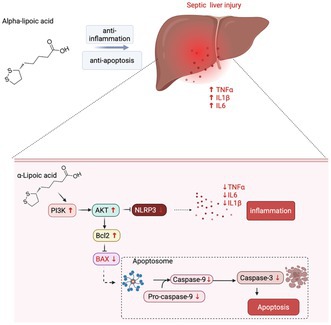

## 1 Introduction

Sepsis is a life‐threatening condition characterized by a dysregulated host response to infection, leading to systemic inflammation and potential organ dysfunction, with the liver being one of the commonly affected organs (Feng et al. 2020; Ford et al. 2025). Cecum ligation and puncture (CLP) is now a recognized method for modeling abdominal sepsis (Dejager et al. 2011). Peritoneal sepsis, typically caused by bacterial or yeast peritonitis, results in the release of large quantities of pro‐inflammatory cytokines, which can trigger liver injury (Lelubre and Vincent 2018). Despite advances in medical technology, sepsis remains a significant cause of morbidity and mortality, and the currently available treatments are relatively limited (Antcliffe et al. 2025; Thwaites et al. 2025).

The treatment of sepsis primarily involves early detection, source control, and supportive care, such as the use of antibiotics and maintenance of hemodynamic stability (Li et al. 2025; Giamarellos‐Bourboulis et al. 2024). However, there are fewer specific pharmacological interventions for sepsis‐associated liver injury, highlighting the urgent need for the development of novel therapeutic strategies.

Sepsis‐related liver injury (SRI) is a major complication, contributing to multiorgan dysfunction and poor prognosis (Strnad et al. 2017). The liver, vital in immune regulation and metabolism, is particularly susceptible during sepsis, with injury characterized by hepatocyte apoptosis, necrosis, and impaired function, potentially leading to acute liver failure. The pathophysiology involves inflammatory cytokines, oxidative stress, mitochondrial dysfunction, and altered hemodynamics, worsening liver damage (Lu et al. 2022). Current treatments are supportive, targeting infection and hemodynamic status, but no specific therapies exist to directly treat SRI. Although antioxidants and anti‐inflammatory agents show promise in preclinical studies, their clinical effectiveness remains limited (Huo et al. 2023).

Alpha‐lipoic acid (ALA) is a natural antioxidant that shows promise in protecting the liver from a wide range of injuries (Superti and Russo 2024; Vafaee et al. 2025). ALA is a compound with a wide range of bioactivities that have broad effects on systemic health. In the cardiovascular system, ALA can improve lipid levels, lower blood pressure, and prevent thrombosis (Vafaee et al. 2025; Yan et al. 2024). Additionally, ALA can lead to a significant increase in the body's immunity, helping to prevent common illnesses such as colds and flu, and reducing other complications arising from them (Fasipe et al. 2023). It has been confirmed that ALA shows potential anti‐inflammatory activity, inhibiting nitric oxide (NO) and prostaglandin E2 (PGE2) production by lipopolysaccharide (LPS)‐stimulated macrophages, and significantly reducing the mRNA levels and protein expression of inducible nitric oxide synthase (iNOS), cyclooxygenase 2 (COX‐2), and tumor necrosis factor (TNF‐α) (Ruchika et al. 2024). This anti‐inflammatory effect helps to reduce the inflammatory response and alleviate the symptoms of related diseases. ALA also protects the nervous system, lowers blood sugar, and contributes to anticancer therapy (de Sousa et al. 2019). Studies have shown that ALA can reduce liver injury by reducing oxidative stress and modulating the inflammatory response (Yu et al. 2023).

However, the protective effects of ALA on the liver in sepsis are currently understudied. Despite the current research base, further studies are needed to fully elucidate the specific mechanisms by which ALA exerts its protective effects in sepsis‐induced liver injury and to determine its efficacy in clinical settings. Nevertheless, the available evidence suggests that ALA has the potential to become an important drug for the treatment of sepsis‐related hepatic complications, providing new ideas and options for clinical treatment.

## 2 Methods

### 2.1 Animals

Male C57BL/6 mice (6–8 weeks old, 20–25 g) were purchased from Liaoning Changsheng Biological Center, Shenyang, China, and housed under controlled conditions (24°C ± 2°C, 50%–70% humidity, 12‐h light/dark cycle). Mice were fed standard chow and water ad libitum. All experiments followed ethical guidelines approved by the Ethics and Animal Care Committee of China Medical University. Mice were randomly assigned to four groups: sham, CLP, CLP + ALA (50 mg/kg, 100 mg/kg). ALA was administered by gavage 2 h prior to CLP. The CLP model was used to induce polymicrobial sepsis, mimicking human sepsis. Mice were anesthetized (ketamine and xylazine), and the abdomen was sterilized and incised. The cecum was exteriorized, punctured with a 22‐gauge needle, and contents extruded to ensure contamination. The abdomen was sutured, and mice received 50 mL/kg saline for resuscitation. Sham surgeries involved no cecal ligation or puncture. All procedures were performed by a single surgeon within 10 min. Samples were collected 24 h post‐CLP. Survival experiments were observed until 48 h postoperatively.

### 2.2 Western Blot

Protein samples were processed following the methods outlined in the earlier study. Equal quantities of protein were resolved on 7.5%–12.5% sodium dodecyl sulfate‐polyacrylamide gels and transferred onto polyvinylidene fluoride membranes (Millipore Corp., Bedford, MA, USA). The membranes were blocked with 5% skim milk at room temperature for 1 h and then incubated overnight at 4°C with primary antibodies specific for CD86 (rabbit, 1:1000, Abcam, ab317266), CD206 (rabbit, 1;1000, Abcam, ab252921), CD45 (rabbit, 1:500, Cell Signaling, Cat. #70257S), IL‐6 (rabbit, 1:1000, Wanleibio, WL02841), TNF‐α (rabbit, 1:1000, Wanleibio, WL01581), p‐AKT (rabbit, 1:1000, Cell Signaling, Cat. #9271S), AKT (rabbit, 1:1000, Cell Signaling, Cat. #9272), p‐PI3K (rabbit, 1:1000, Cell Signaling, Cat. #4292S), PI3K (rabbit, 1:1000, Cell Signaling, Cat. #4292), caspase‐9 (rabbit, 1:1000, Cell Signaling, Cat. #9502), caspase‐3 (rabbit, 1:1000, Cell Signaling, Cat. #9662), Bax (rabbit, 1:1000, Proteintech, Cat. 50,599‐2‐Ig), Bcl‐2 (rabbit, 1:1000, Proteintech, Cat. 60,178‐1‐Ig), and GAPDH (mouse, 1:5000, Proteintech, 60,004‐1‐Ig). Following this, the membranes were incubated with secondary antibodies for 1 h, and the resulting ECL western blot images were captured and analyzed using a chemiluminescence imaging system (Biorad, USA).

### 2.3 Real‐Time Polymerase Chain Reaction (RT–PCR)

Total RNA was extracted from tissues using Trizol reagent (Thermo Fisher Scientific, USA), and cDNA was synthesized using the ChamQ Universal SYBR qPCR Master Mix (Vazyme, China). The qPCR protocol consisted of an initial denaturation at 95°C for 30 s, followed by 40 cycles of 95°C for 10 s and 60°C for 30 s. The qPCR was performed using the SYBR‐Green System (Yeasen, Shanghai) according to the manufacturer's instructions. Relative gene expression levels were quantified using the 2^−ΔΔCt^ method, with GAPDH serving as the internal control and reference gene. The primers used in this study are listed in Table 1.

**TABLE 1:** Primers and sequences used in the study.

	Forward primer	Reverse primer
*Col1a1*	GCTCCTCTTAGGGGCCACT	CCACGTCTCACCATTGGGG
*Col3a1*	CTGTAACATGGAAACTGGGGAAA	CCATAGCTGAACTGAAAACCACC
*Col4a1*	CTGGCACAAAAGGGACGAG	ACGTGGCCGAGAATTTCACC
*IFN‐a*	TGTCTGATGCAGCAGGTGG	AAGACAGGGCTCTCCAGAC
*IL‐1b*	GCAACTGTTCCTGAACTCAACT	ATCTTTTGGGGTCCGTCAACT
*IL‐17*	TTTAACTCCCTTGGCGCAAAA	CTTTCCCTCCGCATTGACAC
*AKT1*	ATGAACGACGTAGCCATTGTG	TTGTAGCCAATAAAGGTGCCAT
*STAT3*	CAATACCATTGACCTGCCGAT	GAGCGACTCAAACTGCCCT
*BCL2*	GTCGCTACCGTCGTGACTTC	CAGACATGCACCTACCCAGC
*MMP9*	CTGGACAGCCAGACACTAAAG	CTCGCGGCAAGTCTTCAGAG
*CASP3*	ATGGAGAACAACAAAACCTCAGT	TTGCTCCCATGTATGGTCTTTAC
*GAPDH*	AGGTCGGTGTGAACGGATTTG	TGTAGACCATGTAGTTGAGGTCA

### 2.4 Hematoxylin Eosinstaining (HE) Staining

Paraffin‐embedded mouse liver tissue sections were deparaffinized and hydrated. The sections were stained with hematoxylin, rinsed with water, and then treated with dilute hydrochloric acid in alcohol before being stained with eosin. The tissue was dehydrated using increasing alcohol concentrations. Finally, the sections were observed under a light microscope.

### 2.5 Immunohistochemical

5 μm paraffin tissue sections were heated at 65°C for 45 min, followed by deparaffinization and rehydration. Antigen retrieval was performed in citrate buffer (pH 6.0) using an autoclave. The sections were incubated in 3% hydrogen peroxide for 15 min, rinsed three times with PBS, and then incubated with 5% BSA at room temperature for 30 min. Next, the sections were incubated overnight at 4°C with monoclonal antibodies against NLRP3 (rabbit, 1:700, Immunoway, YT5382), CD86 (rabbit, Abcam, ab317266,1:700), CD45 (rabbit, 1:500, Cell Signaling, Cat. #70257S). After rinsing with PBS (three times for 5 min each), the sections were incubated with polymerized horseradish peroxidase‐labeled goat anti‐mouse IgG (1:1000, Beyotime) and goat anti‐rabbit IgG (1:1000, Beyotime) at 37°C for 40 min. Detection was carried out using DAB staining (Vector Labs), and the sections were then counterstained with hematoxylin. Each experiment included positive and negative controls. Finally, the sections were dehydrated, cleared, and mounted with neutral gel.

### 2.6 Sirius Red Staining

Liver tissue sections (5 μm, paraffin‐embedded) were deparaffinized in xylene for 15 min, then rehydrated through graded ethanol to ddH2O. Sirius Red stain was applied for 30 min at room temperature, followed by a 5‐min rinse with water. The sections were dehydrated through ethanol, each for 2 min, then cleared in xylene for 5 min before mounting coverslips. The Modified Kit (No Picric Acid, G1472) was from Slarbio.

### 2.7 Network Pharmacology Research

ALA's chemical structure was converted to SDF and SMILES formats using PubChem. Potential sepsis‐related targets were identified with SuperPred, Swiss Target Prediction, and PharmMapper (score ≥ 4.0). These were validated with STRING and cross‐referenced with sepsis‐related targets from Gene Cards, TTD, and OMIM. Nonduplicate targets were considered significant, with drug and disease data sourced from UniProt. Shared targets between ALA and sepsis were identified using Venny 2.1 and imported into STRING (confidence threshold > 0.4). The PPI network was visualized using Cytoscape.

### 2.8 Flow Cytometry

Liver and spleen tissues were excised, and mononuclear cells were isolated using Trizol reagent. The cells were fixed, washed, and stained. For apoptosis analysis, cells were incubated with a staining solution for 5 min, followed by the addition of Annexin V‐FITC‐IgG antibody. After thorough mixing, an equal volume of staining solution was added. Flow cytometry was used to measure fluorescence intensity, enabling the analysis of apoptotic cell populations in both tissues.

### 2.9 Statistical Analyses

GraphPad Prism 10 software was used to conduct all statistical analyses. Continuous variables were presented as means ± SEM. For behavioral tests, group differences were assessed using two‐way repeated‐measures analysis of variance (ANOVA) followed by Bonferroni's post hoc test. Immunofluorescence, RT‐PCR, and western blotting data were analyzed using one‐way ANOVA with Tukey's post hoc test for intergroup comparisons. A significance level of *p* < 0.05 was considered statistically significant for all analyses.

## 3 Results

### 3.1 ALA Treatment Improved Survival and Reduced Liver Fibrosis in Mice With Sepsis

**FIGURE 1:**
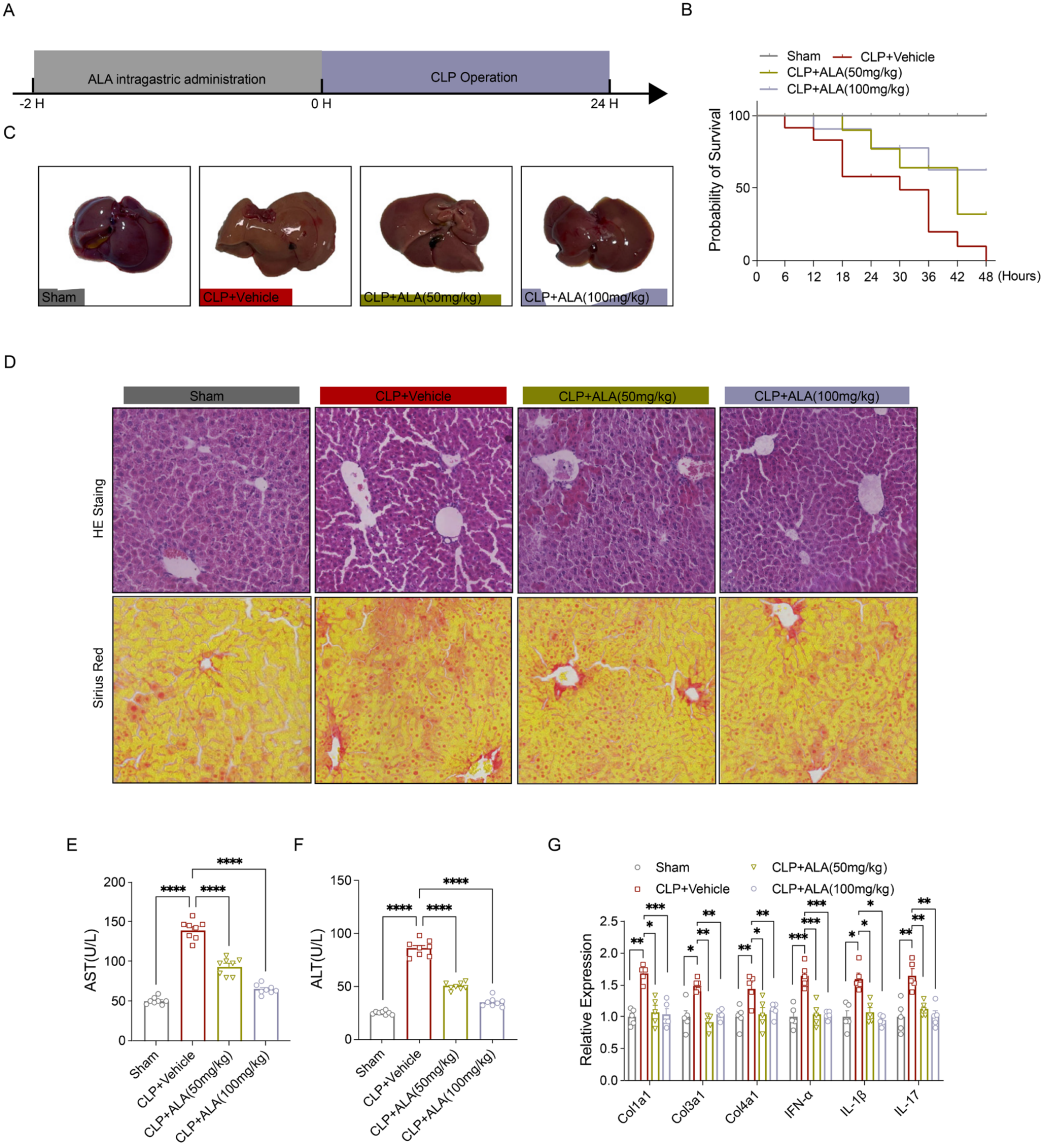
ALA treatment enhanced survival and reduced liver fibrosis in septic mice. (A) ALA was administered via gavage (50, 100 mg/kg) 2 h prior to CLP. (B) Postoperative survival rates at 48 h for the Sham, CLP + Vehicle, and CLP+ ALA (50, 100 mg/kg) groups (*n* = 8). (C) Representative images of liver bulk from the three groups. (D) HE and Sirius Red staining of liver tissue (scale bar, 50 μm). (E) Comparative analysis of serum ALT levels (U/L). (F) Comparative analysis of serum AST levels (U/L) (*n* = 8). (G) Relative mRNA expression levels of fibrosis‐related genes (*Col1a1*, *Col3a1*, *Col4a1*, *IFN‐α*, *IL‐1β*, *IL‐17*) in liver tissues (*n* = 5). Data are presented as mean ± SEM. *p < 0.05, **p < 0.01, ***p < 0.001, ****p < 0.0001 indicate statistically significant differences.

Mice were administered ALA at doses of 50 and 100 mg/kg, respectively, 2 h prior to undergoing CLP surgery (Figure 1A). Between 6‐and 12‐h post‐CLP, the majority of animals exhibited systemic disease symptoms, including lethargy, pronounced dyspnea, and piloerection. The 24‐h survival rate was 0.615 (8/13) in the CLP group and 0.154 (2/13) in the 48‐h group. In contrast, the survival rate was 0.692 (9/13) in the 50 mg/kg ALA group and 0.769 (10/13) in the 100 mg/kg ALA group within the 48‐h group (Figure 1B). Histological analysis via hematoxylin and eosin (HE) staining revealed that liver tissue in the CLP group exhibited significant pathological changes, including inflammatory cell infiltration, hepatocyte swelling, and cytoplasmic vacuolization. ALA treatment significantly mitigated these histopathological alterations caused by sepsis (Figure 1C). Furthermore, Sirius red staining demonstrated a reduced area of collagen deposition in the liver tissue of ALA‐treated mice compared to that of the CLP group (Figure 1D). Biochemical analysis showed that CLP‐induced sepsis led to significant liver damage, as evidenced by elevated serum levels of alanine aminotransferase (ALT) and aspartate aminotransferase (AST) (Figure 1E,F). ALA treatment notably attenuated the CLP‐induced increases in both ALT and AST levels. Additionally, liver fibrosis‐related factors exhibited analogous changes, with upregulation of mRNA levels observed following CLP (Figure 1G).

### 3.2 ALA Treatment Reduced Liver Inflammation in Mice With Sepsis

**FIGURE 2:**
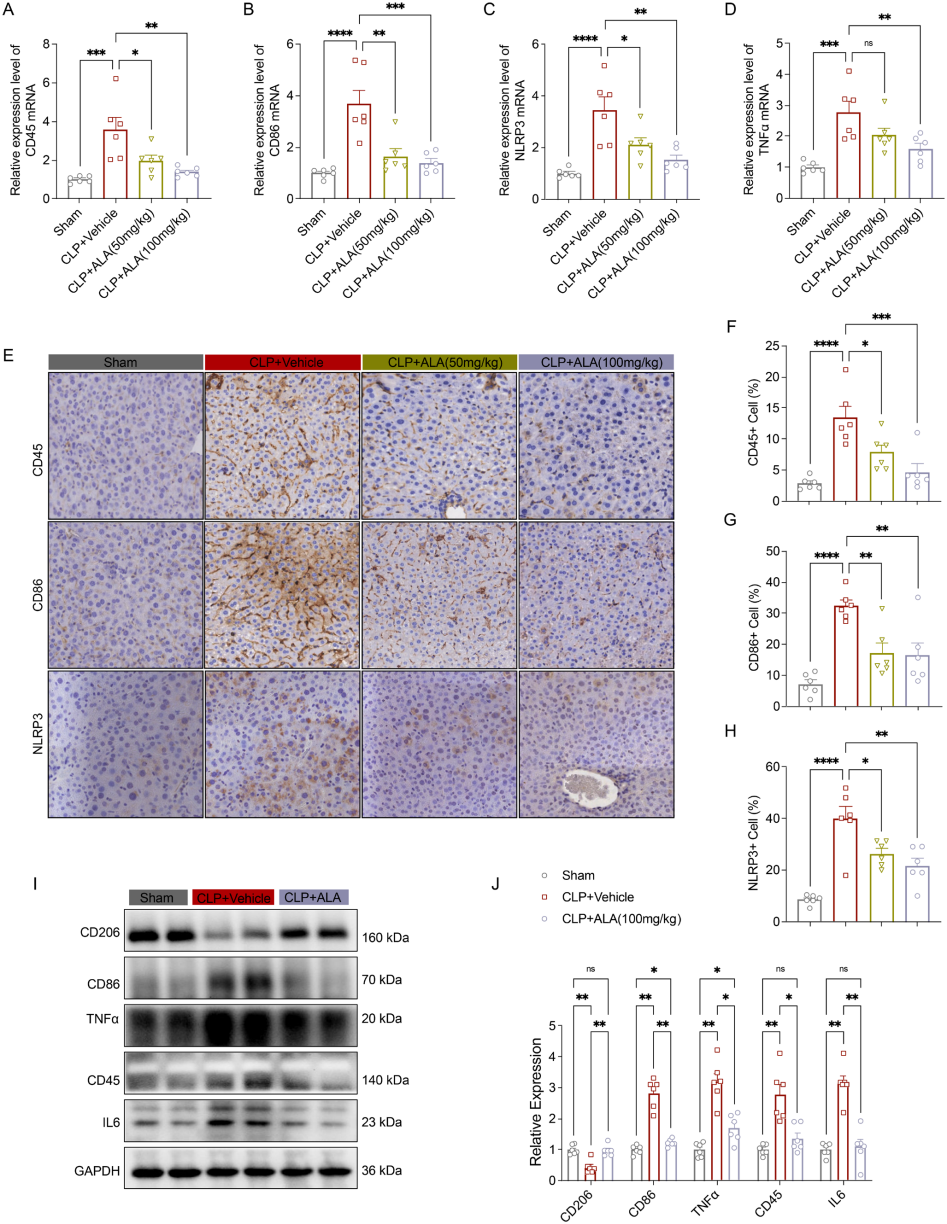
ALA treatment alleviated hepatic inflammation in septic mice. (A) Relative mRNA expression levels of CD45 in liver tissues (*n* = 6). (B) Relative mRNA expression levels of CD86 in liver tissues (*n* = 6). (C) Relative mRNA expression levels of NLRP3 in liver tissues (*n* = 6). (D) Relative mRNA expression levels of TNFα in liver tissues (*n* = 6). (E) Levels of CD45^+^, CD86^+^, and NLRP3^+^ immunohistochemical staining in liver tissue (scale bar = 100 μm). (F–H) Quantitative analysis of CD45^+^, CD86^+^, and NLRP3^+^ in liver tissue (*n* = 8). (I, J) Immunoblotting and relative quantitative analysis of inflammation‐related proteins in liver tissues, including CD206, CD86, TNFα, CD45, and IL6 (*n* = 6). Data are presented as mean ± SEM. *p < 0.05, **p < 0.01, ***p < 0.001, ****p < 0.0001 indicate statistically significant differences.

Numerous studies have demonstrated that abdominal sepsis elicits systemic inflammatory responses, and that intervention with alpha‐lipoic acid (ALA) can effectively alleviate hepatic inflammation. Following cecal ligation and puncture (CLP) surgery, the mRNA expression levels of several intrahepatic pro‐inflammatory factors, including CD45, CD86, NLRP3, and TNF‐α, were significantly upregulated. ALA intervention, however, effectively reversed these changes (Figure 2A–D). Immunohistochemical (IHC) staining further corroborated these findings. The expression of CD45, CD86, and NLRP3 in the liver tissue of the CLP group was significantly elevated, indicating a robust inflammatory response. In contrast, ALA intervention notably reduced the expression of these inflammatory markers, thereby mitigating the inflammatory burden (Figure 2E–H). Moreover, Western blot analysis revealed that CLP surgery enhanced the expression of pro‐inflammatory proteins such as CD86, TNF‐α, CD45, and IL‐6, while simultaneously suppressing the expression of the anti‐inflammatory protein CD206. ALA intervention not only inhibited the upregulation of pro‐inflammatory proteins but also promoted the expression of anti‐inflammatory proteins, thereby restoring a more balanced inflammatory profile (Figure 2I,J).

### 3.3 Network Pharmacology of ALA Against Sepsis

**FIGURE 3:**
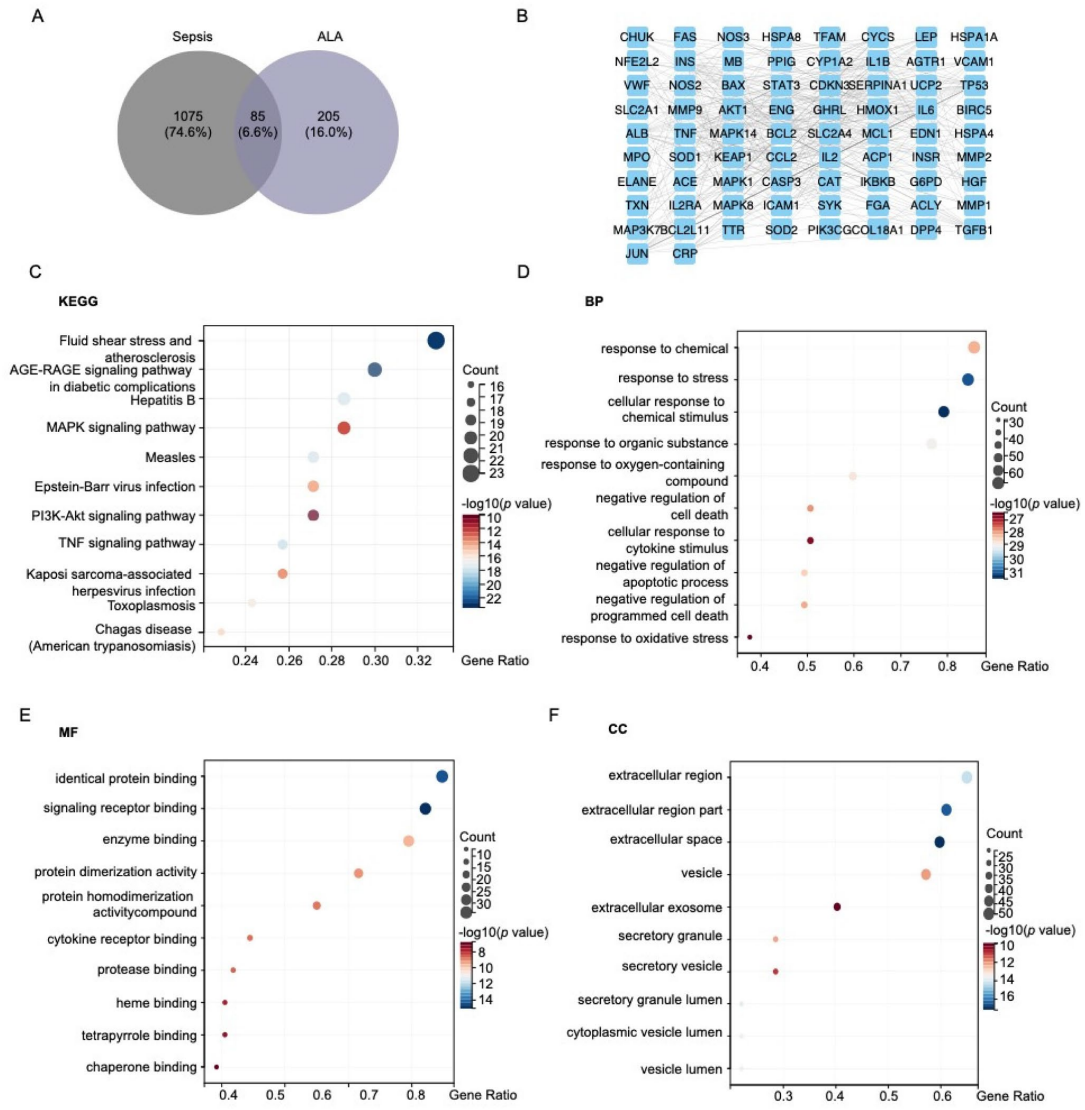
Network pharmacology of ALA regulation of sepsis. (A) Venn diagram of cross‐targets of ALA with sepsis. (B) Pharmacological target of action map of the ALA network. (C) Enrichment analysis of KEGG pathways. (D–F) GO analysis of cross‐targets functional analysis circle diagrams, biological process (BP), molecular function (MF), and cellular component (CC) bubble diagrams. (H) Enrichment analysis of KEGG pathways.

The intersection analysis of 290 predictive proteins associated with alpha‐lipoic acid (ALA) and 1160 proteins implicated in CLP‐induced disease revealed 85 overlapping proteins (Figure 3A,B). These overlapping proteins were further scrutinized to identify key core proteins involved in the interaction between ALA and CLP‐induced pathology. To elucidate the relevant biological processes and pathways associated with these overlapping proteins, Gene Ontology (GO) and Kyoto Encyclopedia of Genes and Genomes (KEGG) pathway analyses were performed. The KEGG analysis identified several key enriched pathways, including the PI3K‐Akt signaling pathway, MAPK signaling pathway, and TNF signaling pathway (Figure 3C). These pathways are known to play crucial roles in regulating inflammation, cell survival, and tissue repair. Additionally, GO functional enrichment analysis was conducted using the DAVID platform, covering three domains: biological process (BP), cellular component (CC), and molecular function (MF). The results indicated that the targets of ALA are involved in a wide array of biological processes and molecular functions. These include cellular response to external stress, response to stimuli, enzyme binding, signal receptor binding, and lipid binding (Figure 3D–F). These findings suggest that ALA may exert its protective effects through multiple mechanisms, targeting various cellular processes and signaling pathways that are dysregulated in CLP‐induced sepsis.

### 3.4 PPI Network and Molecular Docking Analysis of Potential Targets for ALA Treatment of CLP

**FIGURE 4:**
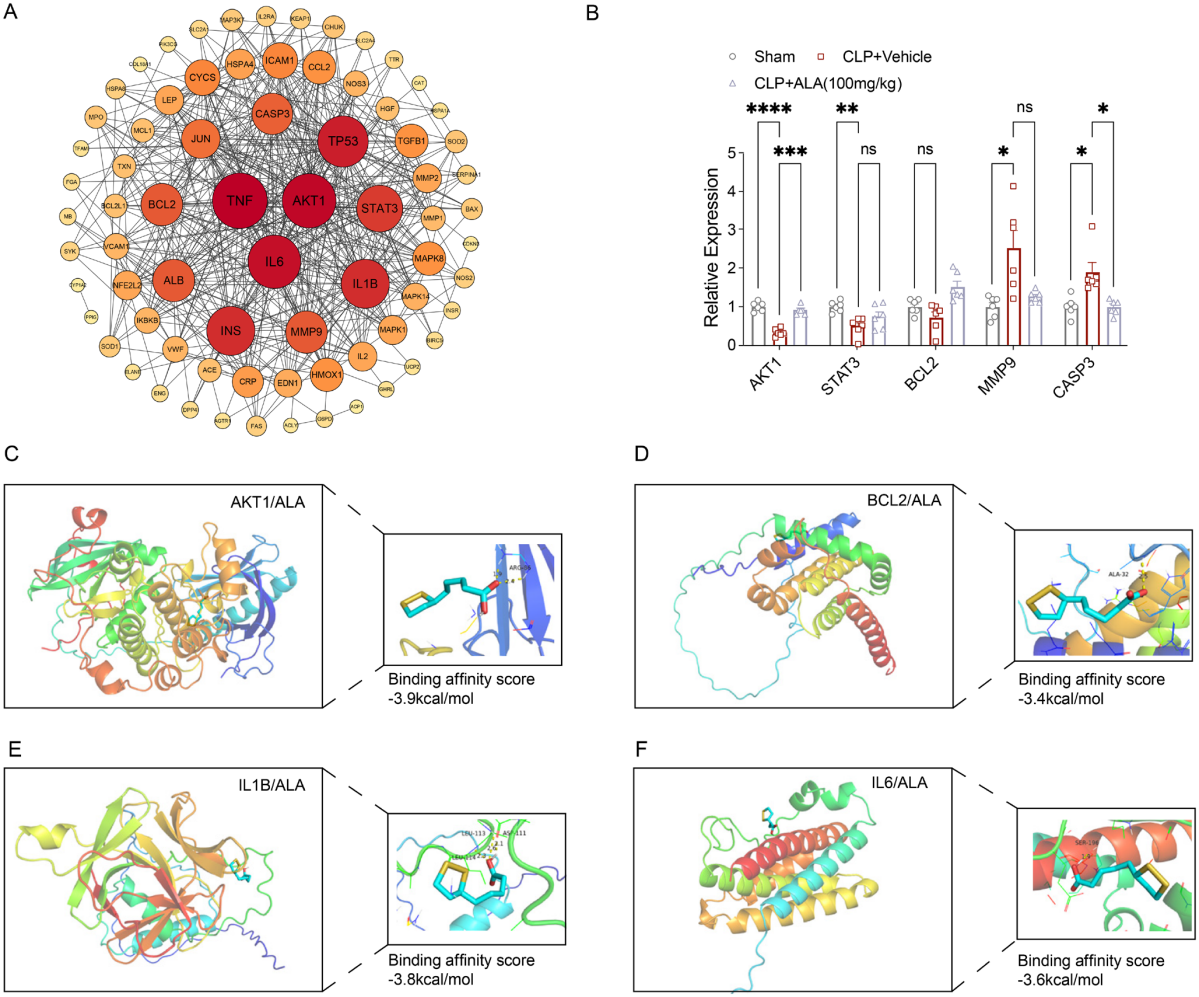
PPI network and molecular docking analysis of ALA. (A) PPI network analysis. (B) Relative mRNA expression levels of target genes (*n* = 6). (C) Protein docking analysis of AKT1 and ALA. (D) Protein docking analysis of BCL2 and ALA. (E) Protein docking analysis of IL1B and ALA. (F) Protein docking analysis of IL6 and ALA. Data are presented as mean ± SEM. *p < 0.05, **p < 0.01, ***p < 0.001, ****p < 0.0001 indicate statistically significant differences.

The top nodes of the PPI network construction analysis were AKT1, TP53, STAT3, BCL2, MMP9, and CASP3 (Figure 4A). Based on the experimental results, we evaluated the mRNA levels in the liver. ALA mitigated the CLP‐induced reduction in AKT1 and BCL2 expression, while inhibiting the upregulation of CASP3 (Figure 4B). Additionally, molecular docking analysis was performed to examine the interactions between ALA and key targets, including AKT1, BCL2, IL1B, and IL6. A binding energy value less than 0 indicates spontaneous binding between the ligand and receptor, with lower values reflecting more stable binding conformations. The molecular docking results revealed that the binding affinities of ALA for AKT1, BCL2, IL1B, and IL6 were −3.9 kcal/mol, −3.4 kcal/mol, −3.8 kcal/mol, and −3.4 kcal/mol, respectively (Figure 4C–F).

### 3.5 ALA Treatment Exerted Anti‐Apoptotic Effects

**FIGURE 5:**
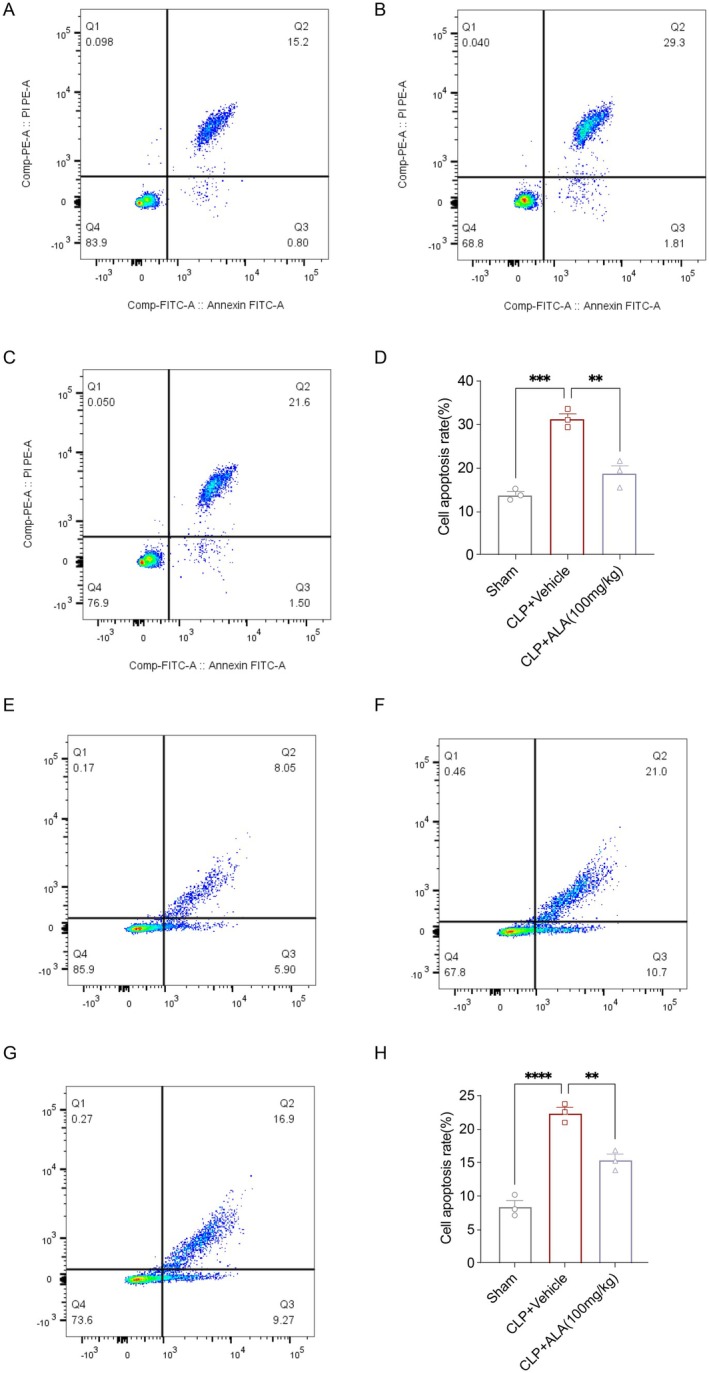
ALA exerted anti‐apoptotic effects in hepatocytes and splenocytes. (A–C) Apoptosis rate of hepatocytes was measured using flow cytometry. (D) Statistical analysis of apoptosis rate. (E–G) Measurement of apoptosis rate of splenocytes using flow cytometry. (H) Statistical analysis of apoptosis rate (*n* = 3). Data are presented as mean ± SEM. *p < 0.05, **p < 0.01, ***p < 0.001, ****p < 0.0001 indicate statistically significant differences.

Network pharmacological analysis and molecular docking results showed that ALA treatment of sepsis was associated with anti‐apoptotic effects. Therefore, we examined liver apoptosis after sepsis using flow cytometry techniques. Apoptosis analysis showed that CLP significantly increased the proportion of apoptotic cells relative to the Sham group. However, ALA administration significantly decreased the proportion of apoptotic cells compared to the CLP group (Figure 5A–D). We also examined apoptosis in the spleen and found that ALA similarly reversed CLP‐induced apoptosis in splenocytes (Figure 5E–H).

### 3.6 ALA Treatment Inhibited Apoptotic Protein Production Through the PI3K/AKT Pathway

**FIGURE 6:**
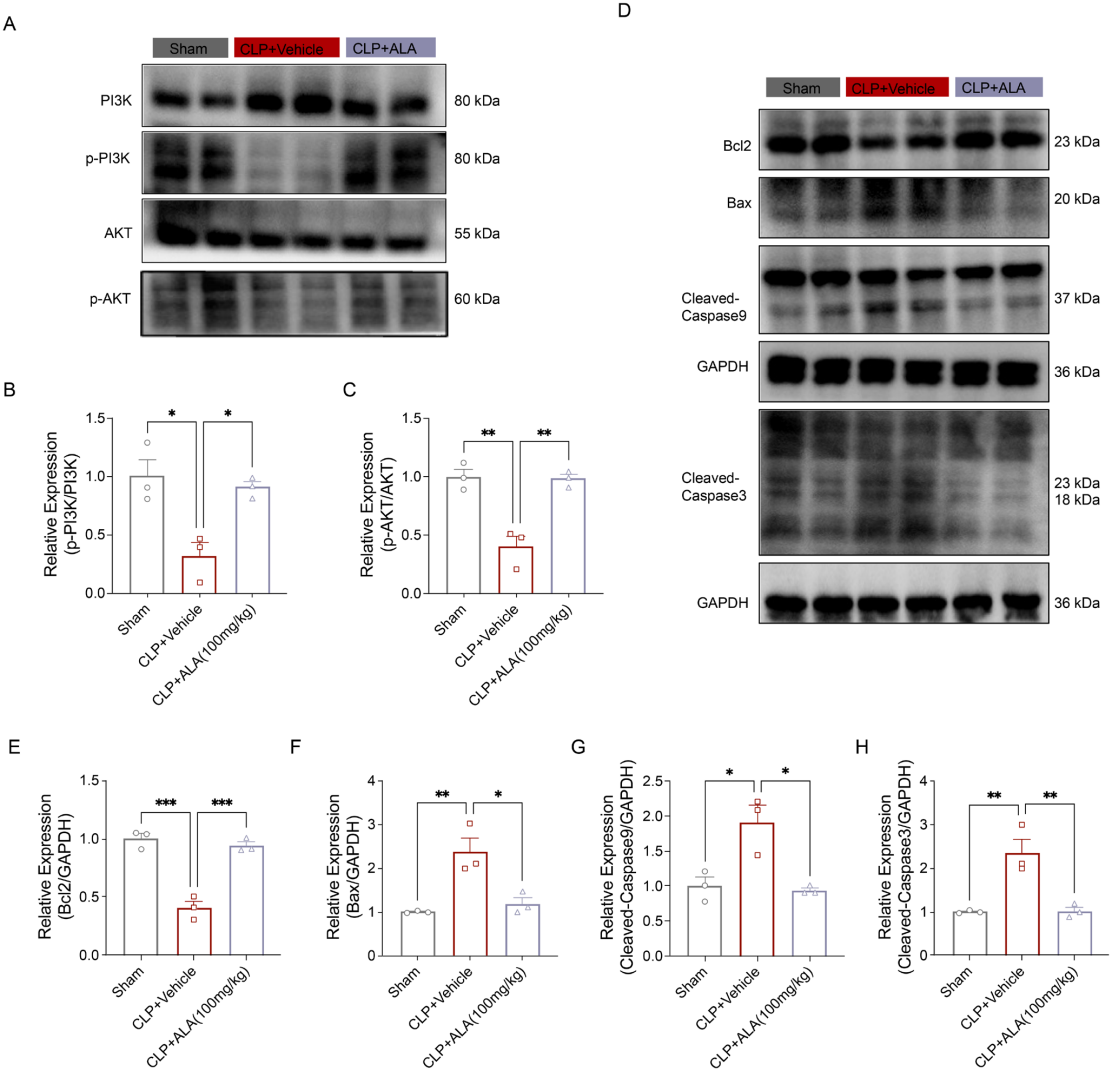
ALA inhibited apoptosis through the PI3K/AKT pathway. (A–C) Immunoblotting and relative quantitative analysis of PI3K, p‐PI3K, AKT, p‐AKT in liver tissues (*n* = 3). (D) Immunoblotting of Bcl2, Bax, Cleaved‐Caspase9, and Cleaved‐Caspase3 in liver tissues (*n* = 3). (E) Relative quantitative analysis of Bcl2. (F) Relative quantitative analysis of Bax. (G) Relative quantitative analysis of Cleaved‐Caspase9. (H) Relative quantitative analysis of Cleaved‐Caspase3 (*n* = 3). Data are presented as mean ± SEM. *p < 0.05, **p < 0.01, ***p < 0.001 indicate statistically significant differences.

Based on the findings from network pharmacology and molecular docking, we further validated these results in the CLP model. WB analysis revealed that ALA treatment significantly enhanced the phosphorylation of PI3K and AKT (Figure 6A–C). Concurrently, ALA treatment promoted the synthesis of BCL2 while inhibiting the expression of BAX. These two proteins, BAX and BCL2, play crucial roles in regulating apoptosis (Figure 6D–F). Additionally, we assessed the caspase family of proteins, which are key mediators of the apoptotic process. In particular, the activation of Caspase‐9 in the apoptotic pathway is typically initiated by the formation of a Caspase‐9 heterodimer. Once this dimer is formed, Caspase‐9 undergoes further activation, which subsequently triggers the activation of downstream effector caspases, such as Caspase‐3 (Figure 6D,G,H). Our results demonstrated that ALA treatment reversed the CLP‐induced activation of both Caspase‐9 and Caspase‐3. These findings suggest that ALA may inhibit the CLP‐induced activation of Caspase‐9 and Caspase‐3 by modulating the PI3K/AKT signaling pathway, thereby mitigating apoptosis during sepsis.

## 4 Discussion

Sepsis is a life‐threatening systemic inflammatory response triggered by bacterial or other pathogenic infections, often accompanied by multiple organ failure, and remains one of the leading causes of mortality among patients in intensive care units (Cajander et al. 2024). The onset and progression of sepsis are intricately linked to a hyperactive immune response, hemodynamic instability, and elevated oxidative stress (Martin‐Loeches et al. 2024). Among the many vital organs affected by sepsis, the liver plays a central role, not only in detoxification and metabolism but also in regulating immune responses (Strnad et al. 2017). Sepsis‐induced systemic inflammation can cause liver cell injury, which is reflected by abnormal liver function, hepatocyte apoptosis, and necrosis, all of which exacerbate the underlying pathology of sepsis (Beyer et al. 2022). Furthermore, liver dysfunction impairs the body's detoxification capabilities, while simultaneously amplifying the systemic inflammatory response, thus creating a vicious cycle. Given this, mitigating liver injury is crucial for improving outcomes in sepsis treatment (Sun et al. 2020).

Alpha‐lipoic acid is a naturally occurring antioxidant widely distributed in living organisms, particularly within cellular mitochondria (Shanaida et al. 2025). ALA is a unique antioxidant because of its dual solubility, which allows it to function both inside and outside of cells. Its key actions include scavenging free radicals, regenerating other antioxidants (such as vitamins C and E), and providing protection against oxidative damage (Wang et al. 2025). Over recent years, the therapeutic potential of ALA has gained attention due to its antioxidant, anti‐inflammatory, and antiaging properties, demonstrating beneficial effects across various diseases. In sepsis, research into ALA's application is increasingly revealing its ability to alleviate pathological damage by modulating immune responses, reducing oxidative stress, and enhancing cellular function—especially in the protection of vital organs such as the liver (Della Giustina et al. 2017).

In our study, we found that administration of ALA at varying doses increased the 48‐h survival rate of mice in a CLP‐induced sepsis model. Notably, ALA treatment significantly mitigated liver histopathological damage and reduced liver inflammation in these mice, as evidenced by the decreased expression of inflammatory proteins such as CD45, CD86, NLRP3, and TNFα. To explore the underlying mechanisms, we employed a network pharmacology approach, constructing a drug‐target‐disease network. This analysis revealed that the therapeutic effects of ALA in sepsis are linked to multiple biological pathways, with the PI3K/AKT signaling pathway being a central component.

The PI3K/AKT pathway is a critical intracellular signaling cascade involved in regulating cell proliferation, survival, metabolism, immune responses, and adaptation to oxidative stress (Sharma et al. 2019; Deng and Zhou 2023). Dysregulation of this pathway during sepsis, often caused by excessive oxidative stress and inflammation, leads to cellular apoptosis, immune dysfunction, and organ failure (Yang et al. 2023). Under normal conditions, the activation of the PI3K/AKT pathway helps prevent apoptosis by modulating various apoptotic proteins, particularly under conditions of oxidative stress or inflammation. However, in sepsis, this pathway can be impaired due to immune cell overactivation and enhanced oxidative stress, which exacerbates cell death and tissue injury.

One of the primary roles of the PI3K/AKT pathway in sepsis is its regulation of apoptosis. AKT activation promotes the expression of antiapoptotic proteins such as BCL2 and inhibits proapoptotic proteins like Bax (Singh et al. 2019; Spitz and Gavathiotis 2022). This regulation maintains mitochondrial stability, prevents the release of cytochrome C, and reduces the activation of caspase‐9 and caspase‐3, which are essential for initiating apoptosis (Kumariya et al. 2021). By controlling these signaling molecules, the PI3K/AKT pathway effectively protects cells from undergoing apoptosis, thus preserving cell viability and function (Sztolsztener and Chabowski 2024; Zhang et al. 2007). In our experiments, we found that ALA enhanced hepatocyte survival and mitigated liver damage and apoptosis induced by sepsis through the activation of the PI3K/AKT pathway. Specifically, ALA treatment led to increased BCL2 expression, which inhibited Bax, caspase‐9, and caspase‐3 activation, further protecting liver cells from apoptosis.

Moreover, ALA enhances the endogenous antioxidant defense system, thereby reducing oxidative stress and inflammation in sepsis. By modulating the PI3K/AKT pathway, ALA appears to alleviate the systemic inflammatory response and improve immune cell function, offering further protection against the deleterious effects of sepsis (Jia et al. 2019). The anti‐apoptotic action of ALA is particularly significant in sepsis therapy, as inhibiting apoptosis not only helps to reduce organ damage but also improves immune function and modulates the inflammatory response.

In this study, we chose oral administration of ALA due to its practical advantages, including ease of use and noninvasive nature, making it more suitable for long‐term management in sepsis patients. However, we acknowledge that the acute nature of sepsis may require more immediate therapeutic intervention, for which intravenous or intramuscular routes could provide faster and more direct effects. While oral administration is effective in many contexts, it may have slower onset and lower bioavailability compared to intravenous or intramuscular administration, which are more suitable for acute settings where rapid drug delivery is crucial. Moreover, we administered ALA 2 h prior to the CLP procedure as a preventive strategy to reduce early oxidative stress and inflammation in sepsis. The preoperative model does not fully explore ALA's therapeutic potential in advanced sepsis. Future studies will investigate ALA administration at different time points, including post‐sepsis onset, to assess its effectiveness as both a preventive and therapeutic agent for sepsis‐related liver injury.

In conclusion, through the PI3K/AKT pathway, ALA exerts multifaceted protective effects at the cellular level, offering a promising therapeutic strategy to attenuate the pathological consequences of sepsis. Its ability to regulate apoptosis, reduce oxidative stress, and modulate the immune response positions ALA as a potential adjunct in sepsis treatment, especially in preventing liver injury and improving survival outcomes.

## Author Contributions

**Yuqing Song:** conceptualization (lead), data curation (lead), formal analysis (lead), methodology (lead), writing – original draft (supporting). **Yuanrong Mao:** data curation (lead), investigation (lead), methodology (lead). **Qingqing Sui:** conceptualization (equal), data curation (equal), methodology (equal), software (equal), writing – original draft (equal). **Zichen Zhao:** methodology (equal), software (equal). **Daosong Dong:** conceptualization (lead), data curation (supporting), investigation (lead), supervision (lead), writing – original draft (lead).

## Conflicts of Interest

The authors declare no conflicts of interest.

## Data Availability

The data that support the findings of this study are available from the corresponding author upon reasonable request.
